# Soporotrichium Gougeroti in New Zealand

**Published:** 1914-10

**Authors:** 


**Soporotrichium Gougeroti in New Zealand.** Sydney T. Chamtaloup, N. Z. Med. Jour., May, 1914, reports four cases affecting the urinary tract and discusses the morphology, etc., extensively. Inferentially, these are the only cases thus far demonstrated in New Zealand.

